# A Methodological Integrated Approach to Analyse Climate Change Effects in Agri-Food Sector: The TIMES Water-Energy-Food Module

**DOI:** 10.3390/ijerph17217703

**Published:** 2020-10-22

**Authors:** Maria Maddalena Tortorella, Senatro Di Leo, Carmelina Cosmi, Patrícia Fortes, Mauro Viccaro, Mario Cozzi, Filomena Pietrapertosa, Monica Salvia, Severino Romano

**Affiliations:** 1 School of Agricultural, Forestry, Food and Environmental Sciences, University of Basilicata, 85100 Potenza, Italy; mauro.viccaro@unibas.it (M.V.); mario.cozzi@unibas.it (M.C.); severino.romano@unibas.it (S.R.); 2 Institute of Methodologies for Environmental Analysis-National Research Council of Italy (CNR-IMAA) C.da S. Loja, 85050 Tito Scalo (PZ), Italy; senatro.dileo@imaa.cnr.it (S.D.L.); carmelina.cosmi@imaa.cnr.it (C.C.); filomena.pietrapertosa@imaa.cnr.it (F.P.); monica.salvia@imaa.cnr.it (M.S.); 3 CENSE (Center for Environmental and Sustainability Research), NOVA School of Science and Technology, NOVA University Lisbon, 2829-516 Caparica, Portugal; p.fs@fct.unl.pt

**Keywords:** Nexus Thinking, IEA-TIMES model, agri-food system, land use, scenario analysis, climate change mitigation, Basilicata region

## Abstract

The European Union’s 2030 climate and energy policy and the 2030 Agenda for Sustainable Development underline the commitment to mitigate climate change and reduce its impacts by supporting sustainable use of resources. This commitment has become stricter in light of the ambitious climate neutrality target set by the European Green Deal for 2050. Water, Energy and Food are the key variables of the “Nexus Thinking” which face the sustainability challenge with a multi-sectoral approach. The aim of the paper is to show the methodological path toward the implementation of an integrated modeling platform based on the Nexus approach and consolidated energy system analysis methods to represent the agri-food system in a circular economy perspective (from the use of water, energy, biomass, and land to food production). The final aim is to support decision-making connected to climate change mitigation. The IEA-The Integrated MARKAL-EFOM System (TIMES) model generator was used to build up the Basilicata Water, Energy and Food model (TIMES-WEF model), which allows users a comprehensive evaluation of the impacts of climate change on the Basilicata agri-food system in terms of land use, yields and water availability and a critical comparison of these indicators in different scenarios. The paper focuses on the construction of the model’s Reference Energy and Material System of the TIMES model, which integrates water and agricultural commodities into the energy framework, and on the results obtained through the calibration of the model β version to statistical data on agricultural activities.

## 1. Introduction

The 2030 Agenda for Sustainable Development identifies 17 Sustainable Development Goals (SDGs) to be achieved by 2030, which aim to encourage a change in the current development model regarding the environmental, economic, and social dimensions [1]. This document represents one of most important global agreement that highlights an integrated and multi-sectoral vision of the different dimensions of sustainable development. It represents an important reference for the nexus approach, addressing the risks and changes associated with the reduced availability of water, energy, and food, in a growing World’s population context (8.6 billion by 2030 and 9.8 billion by 2050) [2]. In addition, the Paris Agreement commits the signatory parties to reduce drastically their greenhouse gas (GHG) emissions and to take urgent actions to combat climate change and its impacts, supporting a transformation of anthropogenic activities toward more sustainable trajectories [3]. As concerns Europe, the need to make production and consumption patterns more sustainable is also emphasized by the European Green Deal, which aims to make Europe climate-neutral in 2050. In view of this ambitious goal, the political objectives and targets set for the period 2021 to 2030 in the EU’s 2030 climate and energy policy framework will be made more ambitious to effectively support the transition to a climate-neutral economy [4] and to implement the Paris agreement commitments.

To reach the policy objectives, it is necessary to implement coordinated actions that can guarantee economic growth and at the same time a drastic reduction of GHG emissions, to mitigate climate change and support environmental protection.

The “Nexus Thinking” and its multi-sector approach are therefore crucial to respond to the sustainability challenge for an effective management of resources in compliance with the SDGs, the Paris Agreement, and the European climate neutrality goal [5]. Concepts of the Nexus Thinking were successfully applied in developing countries [6,7], and currently also to countries with a more advanced economy [8,9,10]. Since 2011, when it was first brought to the attention of the institutions in the opening report of the World Economic Forum [11], the Nexus approach took on a central role as a method for understanding and modeling the complex interactions between the different resource systems (energy, water, food) [12]. It has therefore been successfully used both by academics and international organizations (e.g., The Food and Agriculture Organization of the United Nations (FAO), The United Nations Economic Commission for Europe (UNECE), World Wide Fund for Nature (WWF), etc.). In particular, the Nexus approach aims at managing efficiently water, energy, and food systems as a whole, minimizing potential conflicts and strengthening intersectoral integration in order to guarantee a secure and sustainable use of resources [13]. In fact, the complex interconnections between energy, water and food are difficult to represent due to the numerous variables and phenomena involved. Water affects food production (e.g., crops, livestock) as well as energy production (e.g., hydropower, cooling water). Energy affects food production (e.g., energy for chemical and mineral fertilizers, transportation, and food storage) and also water supply (e.g., water preparation, desalinization, pumping). Agriculture, a major player in food production, is a major user of water (over 70% of all water consumption globally [14]) and energy (about 30% of the total energy demand). It also affects the water sector through land degradation, changes in runoff and disruption of groundwater discharge. On the other hand, the area available for agricultural activities must also compete for a share of electricity generation from fossil and renewable sources, both in terms of the area required for the installation of power plants and the impact of the related activities (e.g., mining, dams and water flow management, biofuel production, etc.). In particular, cultivation of biofuels, which has a high profitability per hectare and, in many cases, benefits of public incentives, causes an excessive exploitation of territories, generating, indirectly, a potential pressure on the prices of food crops and increasing the competition between land use and water consumption to produce biofuels or food. Rulli et al. [15] estimated that the worldwide production of biofuel exploits 4% of the land and water used for agriculture, corresponding to an area sufficient to feed about 280 million people if used to grow food.

In this already complex situation, it is necessary to consider the negative effects of climate change that as known, affects the availability of resources and land, with significant impacts on the water-energy-food system. A recent Intergovernmental Panel on Climate Change (IPCC) Report [16] highlights how climate change increases the rate and extent of ongoing land degradation through two main factors: increased frequency, intensity of heavy rainfall and extreme high-temperature events. Furthermore, global warming will make soil degradation processes more severe in the various geographical areas due to an increased frequency of floods, droughts, cyclones and hurricanes, forest fires and sea levels rise.

The greatest risk in using the nexus approach without considering the data of climate models is to overlook the possible effects of climate change on the balance between the resources involved in the water-energy-food cycle and within their interactions [17].

In fact, both agriculture and energy production are vulnerable to changes in meteorological parameters and to the occurrence of extreme events such as drought and floods [18]. Temperatures and precipitation levels can strongly influence the availability of water, and consequently the production of energy and food. Crop productivity may increase in Northern Europe due to prolonged thawing period opening the possibility for new crops cultivation [19]. In Southern Europe, and in particular in the Mediterranean basin, crop productivity is negatively affected by droughts, heat waves, reduced water availability and other related phenomena such as pest and disease epidemics [20].

From this perspective, the adoption of a holistic approach that allows representing the interrelationships between the three sectors (water, energy, and food) is a priority to encourage a sustainable and efficient use of resources, reduce risks and define effective integrated policies.

Numerous examples in the literature underline the increasing importance of an integrated approach to water-food-energy challenges and different models have been used to assess WEF interactions ranging from economic to technological, and geographic information systems (GIS) tools. In particular, Endo et al. [21] analyzed the water, energy, and food nexus by reviewing 37 projects across different world regions, to highlight the current state of art by investigating the nexus keywords and stakeholders to characterize the specific nexus type.

Haji et al. [22] used a ‘Node’ methodology based on GIS-based approaches, the Analytical Hierarchy Process and resource assessment to evaluate the critical factors that increase the risk in open field farms and to improve water and energy efficiency. In Pakdel et al. [23] a multi-objective optimization of energy and water management based on GAMS software is performed to minimize both energy and freshwater use, introducing the concept of “energy hubs” and validating the interdependency of energy and water structures. In Lee et al. [24] a nexus approach is used to analyze the water-food-energy interconnections and their economic implications in the sugar industry in India. Nie et al. [25] focus on the agricultural system and use a multi-objective procedure and a comprehensive WEF index to select optimal land allocation strategies that can limit stresses in the water-energy-food nexus. Chiodi et al. [26] integrated the energy and agriculture systems into the IEA-ETSAP methodology to individuate the GHG reduction strategies for Ireland.

Many gaps still need to be filled in the operational application of the nexus concept for decision-making. However, Weitz et al. [27] point out that despite the still open questions, “a nexus approach promotes policy coherence through identifying optimal policy mixes and governance arrangements across the water, energy and food sectors”.

These considerations and the relevant examples discussed above, have strengthened our motivation to develop a modeling platform focused on the agricultural system that integrates the nexus concept in a framework typically used to support decision-making when different competing goals must be achieved. The outcome of this research, The Integrated MARKAL-EFOM System (TIMES)-WEF model, will be validated in selected areas of Mediterranean Europe, in order to evaluate the robustness of solutions at different spatial scales, and to perform a joint assessment of the effects of climate change and agricultural policies. The scenario analysis will focus on two IPCC pathways (RCP4.5 and RCP8.5) and multi-level (European Union, national and local) agricultural, energy and climate policies to evaluate their effects in terms of availability of land, water and energy as well as other parameters of interest for the agri-food system (e.g., fertilizers, pesticides, etc.).

The paper is structured as follows: Section 2 presents a literature review of the Nexus modeling approach and its application to the development of the TIMES-WEF model; Section 3 describes data requirement, technical assumptions, the preprocessing procedure for the implementation of the model data input and the calibration to the statistical base year data of the TIMES-WEF model for agriculture; finally, Section 4 concludes with the main outcomes and future development of the model.

## 2. Modeling WEF Nexus

### 2.1. State of Art

The WEF Nexus currently represents the most advanced methodological and operational approach to address the complexity of sustainable development. It aims to overcome the silo vision and evaluate the interdependencies and management of the different sectors (energy, water, food) as an integrated process. In this way, it is possible to highlight how actions in a sector can influence the management of resources, thus avoiding unwanted consequences and exploiting existing potential synergies.

The conceptualization of the WEF Nexus has become increasingly complex, incorporating a plurality of factors and dimensions e.g., environmental, economic, political, and social. Conversely, there has been a slow development of analytical approaches [28,29] and limited use of modeling tools to assess the correlations between water, energy and food and support an integrated decision-making process [30].

Table 1 reports a summary of main methods used in the literature to address the WEF Nexus (are included in the table also the methods that analyze only some of its components).

However, many applications only focus on dual sector interactions, for example water-food or water-energy, thus implementing a fragmented vision of the WEF Nexus [42], or provide a narrow perspective of the interactions between water, energy and food, with a limited ability to capture multi-sectoral interconnections and interdependencies between different systems [43,44].

Few studies are based on innovative methods to quantify the connections and interactions between sectors, in order to better describe the systems included in the WEF Nexus. These are modeling platforms that can support the integration of sectoral models, creating flexible tools that can accommodate new modeling inputs or extensions. In this way, decision support tools are created to combine physical models with scenario analysis, allowing decision makers to compare the impact of different policies or actions on the analyzed system [45,46,47].

Based on the above considerations, the proven validity of the Nexus approach to tackle a multi-objective problem such as an integrated management of energy, water, and soil resources [48] guided our idea to develop an integrated decision support platform, based on existing consolidated models used for policy analysis.

Taking inspiration from the Irish model [26], the TIMES energy models’ generator [38] has been used to model the agri-food system within an energy system analysis approach based on technical engineering and economic analysis to ensure a sustainable management of agricultural resources.

### 2.2. Overview of ETSAP-TIMES

The TIMES (The Integrated MARKAL-EFOM System [38], (developed by the Energy Technology Systems Analysis Program (ETSAP) of the International Energy Agency (IEA), an autonomous intergovernmental organization born with the 1973–1974 oil crisis and based in Paris, France) is a bottom-up model generator, which uses linear-programming to compute a least-cost energy system, optimized according to several user exogenous constraints, over medium to long-term time horizons [38]. It is widely used to represent local, national, and multiregional energy systems and to perform scenario analysis, exploring possible energy futures in relation to environmental and technical constraints, such as policy measures.

The TIMES models are driven by the end-use sector demands (Industry, Residential, Commercial, Transport and Agriculture).

The energy system configuration is optimised to provide the least-cost solution that corresponds to the best allocation of resources and technologies, which fulfil end-use demands and the scenarios’ constraints at the minimum total discounted cost of the system. The optimization of the reference scenario provides the baseline for the comparison of solutions in the alternative scenario analysis.

The TIMES model structure is usually described through the Reference Energy and Materials System (REMS), which describes the entire supply–demand chain, providing an accurate representation of energy flows from supply/conversion technologies to demand processes. It allows representing all the components related to energy production and use, including emissions and materials. The supply chain describes the extraction import/export and secondary production of primary resources (typically energy and materials) whereas the demand chain represents in detail the commodity flows through the network of real or dummy technologies (or processes), (e.g., mining processes, import processes, energy transformation plants, end-use devices). Any item produced or consumed by a certain technology is called “commodity” (e.g., energy carriers, energy services, materials, money flows and emissions).

The key inputs to the TIMES model deals with all specific data that characterize the system under focus: energy demand, primary energy supply (availability of present and future sources), techno-economic factors (technology development and associated costs), environmental variables (e.g., GHG emission factors), and other policy parameters.

This research takes the standard TIMES modeling framework as a starting point to develop a novel model that focus on the agri-food system through a WEF nexus perspective and that can be merged into the general energy modeling framework, exploiting all conversion processes and the end-use sectors related to agriculture.

### 2.3. TIMES-WEF Model

The TIMES-WEF model represents an innovative application of the water-energy-food nexus approach into the ETSAP-TIMES framework where land use is chosen as an independent driving parameter to connect soil availability with input/output commodities. In particular, a land use-driven model allows evaluating directly the effects of climate change and energy-environmental policies in terms of use of resources (energy, water, and land use), agricultural productivity, highlighting the synergies among the different sectors. The land use demand on the whole time horizon represents therefore the “end-use demand” of the energy and material model to be fulfilled at the minimum feasible cost in compliance with the exogenous constraints on resources. The general objective is to ensure an optimal management of the territory, i.e., able to improve the use of endogenous resources, increase the resilience of the agri-food sector to climatic events and facilitate the implementation of agricultural, energy and environmental policies.

**Figure 1:**
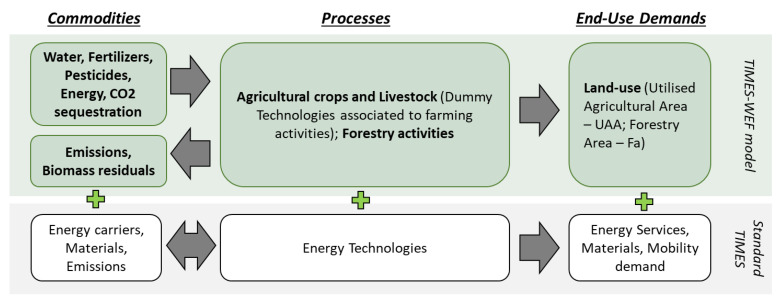
TIMES-WEF flowchart, inspired by Chiodi, 2016 [26].

The analytical structure reported in the flowchart represented in Figure 1 was therefore designed to represent the agri-food system and characterize its data input.

In this modeling approach, the Used Agriculture Area (UAA) and the Forestry Area (FA) represent the output commodities. More precisely, the UAA refers to the total area (hectares) used for agriculture, which includes arable land, permanent meadows, permanent crops, and vegetable gardens used by farms with reference to Eurostat data [49]. The FA, instead, is representative of the hectares of surface area covered by forests or the canopy of the forest or open woods.

The agricultural and forestry activities are modeled as end-use processes (dummy processes) with associated input and output commodities, operating costs, and other key parameters characterizing these practices. New elements such as water, fertilizers, pesticides, and CO_2_ capture from forestry were included among the input commodities of a standard TIMES model (energy vectors and materials). Biomass residuals from agriculture and forestry, greenhouse gas emissions from both the combustion processes and agricultural activities are modeled as process outputs.

The characterization of agricultural activities was defined according to the standard classification of farming classes used in the main European and national databases. Specifically, 10 categories were considered: arable crops, horticulture, viticulture, olive growing, fruit growing, herbivores livestock, granivorous livestock, polyculture, mixed livestock and mixed (that include livestock and crops).

Each agricultural activity was represented by two processes in series: (i) The first process consumes water, energy (electricity, diesel, natural gas), pesticides and/or fertilizers and produces crops (expressed in ton) or cattle (expressed in livestock unit (LSU)); (ii) The second process converts the productions of the first process in hectares of used agricultural/livestock area through a yield parameter. The sum of the outputs of the second processes of all ten categories of agricultural activities provides the demand for end use, i.e., the Used Agriculture Area. The only exception is represented by mixed activities that were modeled as a single process. It has energy and water consumption as input and hectares used as output.

Forests play a multifunctional role, contributing to the protection of biodiversity and the environment (through carbon sequestration) and to the economy (through the production of biomass as an energy resource). They are also particularly affected by climate change (droughts, forest fires, etc.), which reduce their carbon sequestration power and bioenergy resource potential.

**Figure 2:**
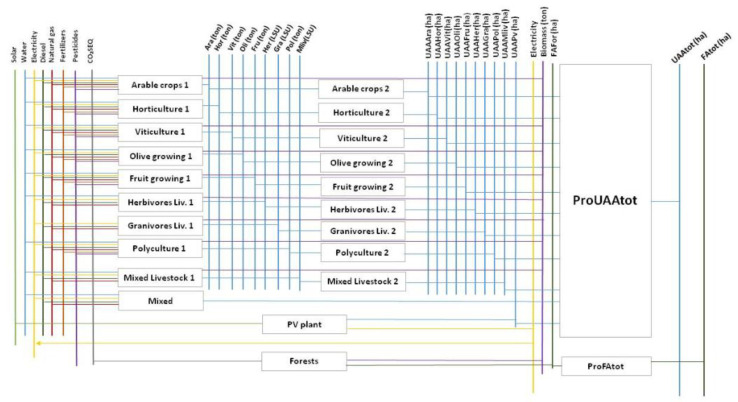
The TIMES-WEF Reference Energy and Materials System (REMS).

The detailed structure of the TIMES-WEF module is described by the flowchart in Figure 2 that shows the flows of commodities through processes, from resource mining to end-use demands (i.e., UAA and FA).

The analyzed time horizon covers a period of 50 years, from 2010, the model base year, to 2060 and was divided into time intervals of 5-years each (model’s time slices), considering 2030 as a milestone. This long-term time horizon makes it possible to evaluate the effects of the strategies for achieving the targets of the 2030 Agenda beyond the year 2030 and to trace the path toward the Energy Roadmap 2050 through a scenario analysis. Furthermore, from a modeling point of view, the choice of a time horizon that goes beyond 2030 derives from the need to harmonize the timing of the TIMES-WEF to the TIMES Basilicata energy system module [50] to allow its integration and increase the reliability of the solutions in a long-term perspective. Therefore, in the future stages, the integration of the TIMES-WEF agri-food model into the whole energy system model, will make possible to have a comprehensive perspective of the synergies and competition between the two sectors in a circular economy perspective.

The TIMES-WEF module data input is made up of a set of an Excel spreadsheet, structured around three type of file:

▪ The Base Year Templates: they contain the basic data about input commodities (energy, water, fertilizers), operating costs and output commodities (products and by-products-including straw, manure and emissions, and Used Agricultural Area) in order to characterize the Forestry and Agricultural Activities in the base year. They provide the statistical data for the model calibration.
▪ A “Technology Repository”: that is a virtual basket of alternative options for agricultural practices, described by technical (e.g., efficiencies, lifetime, emission factors) and economic parameters (e.g., investment and operation costs). These options can be implemented over the time horizon to replace the current processes to fulfill the exogenous constraints.
▪ The Scenario Files: a set of spreadsheets containing coherent demand projections, exogenous constraints on resources availability, and other parameters by scenario.

### 2.4. The Basilicata Region Case Study

To test the applicability and consistency of the integrated approach developed through the TIMES-WEF modeling module, the TIMES model was customized according to the Basilicata region data as a pilot case. Basilicata is a small region located in the South of Italy bordering with Campania on the West, Apulia on the North and East, and Calabria on the South. The region covers 10,073 square kilometers and has a population of 562,869 inhabitants [51] with a quite low regional population density (57.8 inhabitants per km^2^).

Basilicata is a relevant case study mainly due to the urgent threat that the effects of climate crisis represent for its territory. In fact, 55% of the Region is at risk of desertification [52] endangering the future of the agricultural sector, which plays a crucial role in the local economy. Therefore, for this Region it is a priority to define policy mitigation and adaptation actions to increase the resilience of the territory and the agriculture sector, identifying sustainable pathways of local resources. Due to the wide morphological difference, mainly in elevation, the Region is characterized by a varied climate that ranges from the continental one in the internal areas to the Mediterranean one of the coastal areas. There are six distinct soil and climatic zonas (Ionian, Bradanica, Northern Apennines, North Western Apennines and South Western Apennines, Tyrrhenian) in which climate deeply influences the type of agriculture, in particular in the internal and non-irrigated areas [53].

The territory is mainly mountainous (47%) and hilly (45%) with a modest flat percentage (8%). The total agricultural area is 716,838 hectares accounting for about 70% of the regional surface area. The forest area according to the Regional Forest Charter is 355,409 hectares, characterized by variegated species in both environmental and vegetation terms which make the regional territory a mosaic landscape.

As happens in several Mediterranean countries under the pressure of contingent factors (climate change, changes in land use, over-exploitation of resources, etc.), large areas of Basilicata are particularly susceptible to land degradation [54,55,56]. This situation highlights the importance of a more sustainable management of resources and forests to improve the resilience of the territory and guarantee the functioning of a key sector such as agriculture [57]. Therefore, it is a priority for this region to define comprehensive mitigation and adaptation actions that increase the resilience of the territory and to identify sustainable pathways in the use of local resources aimed at improving the agricultural sector. Agriculture is, in fact, one of main activities of the Basilicata economy [58], along with industry (manufacturing, automotive, and especially oil extraction) and services, where the importance of tourism is increasing. Despite its small contribution compared to other sectors, with an added value of 3% in 2019, the agricultural sector has a significant weight in terms of exports and employment, registering for the latter, a positive trend in recent years reaching an increase of 7% in 2019, in contrast with the other sectors [59]. Therefore, the high level of specialization achieved in the agri-food sector (characterized by the production of a wide range of high quality food (most of which included in national list of Protected Designation of Origin (PDO), Protected Geographical Indication (PGI), and Controlled and Guaranteed Designation of Origin (DOCG, the Italian acronym) marks of traditional food and wine products) could be a potential strength to increase the competitiveness of the entire regional system. From this perspective, the 2014–2020 Rural Development Program played a key role by providing a valuable financial support (680 M€) to encourage innovation in this sector in order to improve its economic and sustainability environmental performance [60].

As regard energy resources, the Basilicata region hosts the Europe’s largest onshore oil and gas field, with an annual production of 48,550,554,911 BBOE of oil and 1,493,816,334 Smc of natural gas [61]. Furthermore, Basilicata achieved remarkable targets in the production of renewable electricity, reaching and encompassing the goals set by the Regional Environmental Energy Plan (PIEAR) for 2020, namely 981 MW of onshore wind (60% of the total renewable capacity), 359 MW of solar-photovoltaic (20%), 50 MW of biomass (15%) and 48 MW of hydroelectric (5%). The regional authorities, through various policy measures, have also encouraged the use of biomass to produce thermal energy. In 2017, 45% of the regional energy requirement (thermal and electric) was met by renewable sources, also in this case anticipating the objective of the national legislation (33%) expected by 2020 [62].

The information necessary to implement the TIMES-WEF module for the Basilicata case study was collected by elaborating the national and European statistical sources of data (FADN, RICA and ISTAT), the Regional Environmental Energy Plan–PIEAR and other local sources (e.g., irrigation water, fertilizers and agricultural diesel prices for which there are different values depending on the region) (Table 2).

### 2.5. Scenarios

The scenario analysis has two main objectives in this research: (I) evaluating the effects of climate change in terms of land use variations and resources availability (water and land); and (II) assessing the consequences of EU, national and regional environmental and agricultural policies on the entire agri-food system of Basilicata region.

A reference Business-as-Usual (BaU) scenario was set to provide a benchmark for comparing the alternative scenarios solutions. It represents the development of the system under the policies in force, both energy (through Regional Environmental Energy Policy Plan-PIEAR), and agricultural. The reference scenario shows the “status quo” evolution of the Basilicata Region agricultural system in term of land use availability, resources, technologies, and policy in place. The land use demand by category is projected along the analyzed time horizon (2010–2060) using appropriate statistical techniques.

The selection of scenarios considers the phenomena in progress and the most urgent challenges to be faced in the Mediterranean Europe region for mitigation and adaptation to climate change by focusing on the agri-food sector.

In fact, this area is significantly affected by climate change, especially agriculture. The intensity and frequency of extreme weather event (in particular, heat waves, flooding, wildfires) accelerate the degradation of agricultural land and cause a substantial decrease in yields, with an estimated average loss of 3.24 ton/ha per year compared to 2010 (reference year) [71]. As for the consequences in terms of WEF Nexus, droughts will favor a greater demand for water, with an increase predicted by the Representative Concentration Pathways (RCP) 4.5 and 8.5 climate scenarios between 4% and 18% by the end of the century. This will inevitably reduce the availability of water for irrigation, undermining the suitability of the land for rain crop production [72].

Indeed, through more sustainable agricultural and forestry practices it is possible to reduce the environmental impact of this sector, strengthen the carbon capture capacity of soils and forests and protect biodiversity.

From this perspective, the agriculture and forestry sector has been included for the first time in the greenhouse gas emission reduction targets set by the European Union (EU) for 2030 [73] and has been at the center of two fundamental strategies of the Green Deal, “Farm to fork” and “Biodiversity 2030” [74].

As highlighted by Nikolakopoulou [75] “food related targets run throughout the Sustainable Development Goals (SDG) and they are often interconnected”. In particular, a sustainable agriculture system, in line with the SDGs, should be more resilient to climate risks, protect the environment and deliver healthy and affordable products to fulfill the demand of an increasing population. The new European Green Deal package of measures will have a strong impact on future planning, reinforcing the orientation already taken by the Common Agricultural Policy (CAP), aimed at favoring a progressive abandonment of intensive agriculture in favor of more sustainable cultivation techniques that preserve the soil quality and fertility by reducing the use of fertilizers and pesticides.

Taking into account the complex environmental and policy framework, two alternative classes of scenarios have been defined as follows:

▪ Climate Scenarios modeling the relationship between climate change and land use. They highlight, on the one hand, how agriculture is affected by climate change, and on the other hand, how crucial is the role of this sector within the greenhouse gas emissions reduction strategies. These scenarios are characterized by water and land availability as quantitative parameters.
▪ Policy Scenarios modeling the EU, national and local policies on energy, environment and agriculture in quantitative terms such as percentage of use of pesticides, fertilizers, area for organic agriculture and GHG emissions reduction targets.

The climate scenarios parameters have been selected coherently with the IPCC 2019 report [16]. Moreover, data of water availability and consumption was taken from the Inter-Sectoral Impact Model Intercomparison Project (ISI-MIP) [76], for the RCP 4.5 and RCP 8.5 climate scenarios.

The scenario analysis will identify sustainable development paths for the agricultural system taking into account the general objectives of the EU Green Deal and the “From Farm to Fork” strategy. In this framework, the contribution of the various alternatives to achieving the SDGs relating to energy, climate and sustainable production and consumption models will be assessed (namely SDG 7 “Clean and accessible energy”, SDG12: “Responsible consumption and production” and SDG 13 “Climate action” [77]).

## 3. Results and Discussions

### 3.1. Data Input Construction

Statistical data pre-processing is a fundamental step for the implementation of the model’s data input.

**Figure 3:**
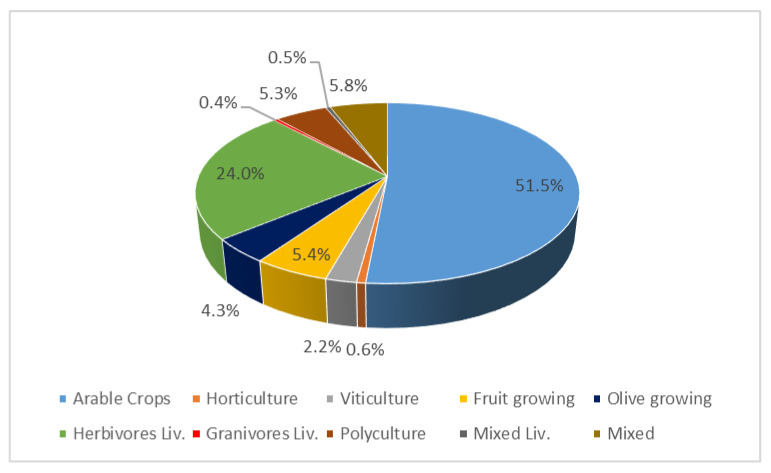
Share of land use by type of farming in Basilicata region.

Following the specifications of the TIMES-WEF model, the agricultural system has been characterized on the basis of the agricultural area (driving parameter), dividing, for the reference year, the total agricultural area in the region (502,197 hectares) into the shares of the various types of farming that characterize the local agricultural system (Figure 3).

The estimations were based on the data of the Agriculture Census for 2010 [63], the latest available survey of the National Statistical Institute, integrated with the information of the annual sample survey of Agricultural Accounting Information Network for Italy (RICA) [65], included in the European Network of Farm Accounting Data (FADN) [78].

The FADN dataset represents the main information source of the European Commission on the Member States to assess the impact of the proposed changes to the CAP through the simulation of different scenarios on corporate sustainability (economic, environmental, social and innovations).

The RICA data were therefore preliminarily processed to represent the agricultural system of the Basilicata region in relation to the 10 categories considered in the model. Further elaborations were also required to model in detail the features of specific sub-categories.

After determining the land use demand by type of crop, the subsequent step was to estimate the energy consumption by type of farming in order to determine the energy balance. This was done considering the Basilicata Regional Environmental Energy Policy Plan (PIEAR) data for Agriculture (Table 3), which was disaggregated through a weighting procedure based on RICA information on the annual energy expenditures for diesel, natural gas and electricity incurred by local farms.

In addition, a further disaggregation was necessary for permanent crops, represented in the TIMES-WEF model by the following categories: viticulture, fruit and olive growing. The energy consumption of each category was estimated by a weighting procedure based on the used agricultural area. Table 4 summarizes the percentage breakdown of the estimated energy consumption for each type of farming.

The costs of the energy carriers for the reference year were estimated considering different sources of data. In particular, the electricity price (59 Euro/MWh, to 16.4 MEuro/PJ) was estimated on the basis of the National Energy Services Operator data [70], diesel price for agriculture (0.60 Euro/liter, 16.2 MEuro/PJ) using the local Chambers of Commerce data [66] and natural gas price (0.66 Euro/liter, 25.3 MEuro/PJ) using the Ministry of Economic Development data [69].

Similarly, the share of water consumption for the ten agricultural activities was calculated using the aggregate data provided by the National Institute of Statistics, assuming a sales price of 0.47 euro per cubic meter [68]. The estimated values by type of crop are shown in Table 5.

The three most important fertilizers used in agricultural practices, potassium (K), phosphorus (P), and nitrogen (N), were also included in the model data input, estimating the tons of fertilizers used in the base year 2010 [65] starting from the hectares of agricultural area for each type of crop and the tons of product (Table 6 and Table 7).

The average costs of fertilizers were estimated based on the Turin Chamber of Commerce data [67] assuming a constant value at national level. Specifically, 359 Euro/ton for Nitrogen, 328 Euro/ton for Phosphorus and 498 Euro/ton for Potassium were considered.

Once characterized the input commodities (diesel, natural gas, electricity, water, and fertilizers), it was necessary to characterize the production processes as fictitious technologies (dummy processes) estimating the operating costs, since the technical parameters are not of interest in this approach.

The agricultural production (expressed in tons) and the number of cattle (expressed in in livestock unit-LSU) for 2010 for each type of agricultural activity were estimated by the RICA database. Furthermore, the fixed and variable operating costs incurred by the farms were estimated considering their annual budgets (Table 8).

Another aspect of the setup of the model’s data input concerned waste production that was estimated considering the statistical data relating to the production of straws and manure by crops and livestock type [79], harmonizing these data to the categories of the TIMES–WEF model (Table 9).

In addition, the emissions of the main greenhouse gases (CO_2_, CH_4_ and N_2_O) were estimated by considering the emission factors provided by the United Nations Framework Convention on Climate Change (UNFCCC) database [80]. In the base year calibration of the β version of the TIMES-WEF model, only the energy combustion processes were initially considered. The emissions from processes will be added subsequently.

### 3.2. Base Year Calibration

**Figure 4:**
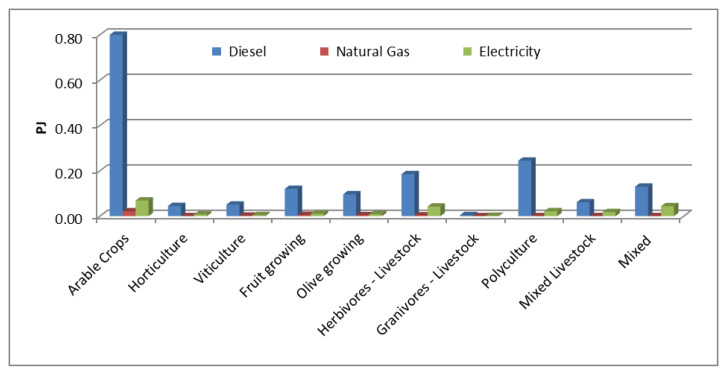
Energy consumption for each type of farming. Base year (2010).

Once the data input for the base year has been implemented, the subsequent fundamental step concerns model calibration to statistics [81]. This is essential to validate the modeling approach and to refine the initial data. The main results of the TIMES-WEF calibration are shown in this section. Figure 4 shows the energy consumption by type of farming. Diesel oil is the most used fuel (1.74 PJ), (in particular from arable crops (0.8 PJ), polyculture (0.25 PJ) and herbivores livestock (0.19 PJ)) followed by electricity (0.23 PJ) and natural gas (0.04 PJ). Granivorous livestock, mixed livestock and mixed farming categories do not use natural gas.

**Figure 5:**
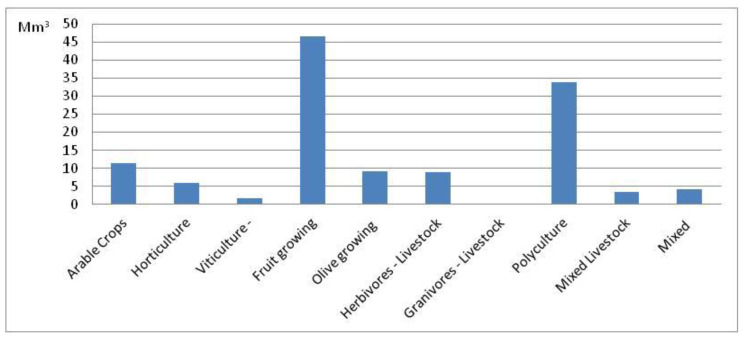
Water consumption by farm typology. Base year (2010).

Figure 5 shows water consumption of the 10 agricultural activities. As expected, fruit growing and polyculture have the highest consumption, 47 Mm^3^ and 34 Mm^3^ respectively, as they are typical “water demanding” categories. Arable crops consumption is about 11 Mm^3^, while granivorous livestock consumption is negligible.

**Figure 6:**
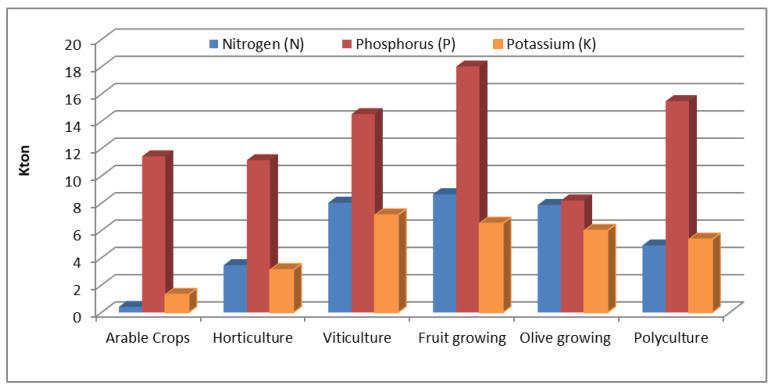
Fertilizers consumption for each type farm. Base year (2010).

As concern the use of fertilizers (Figure 6), fruit growing shows the highest consumption of both Phosphorus (18.02 kton) and Nitrogen (8.66 kton), while viticulture shows the highest consumption of Potassium (14.53 kton). Arable crops, which represent an energy-intensive category covering the highest percentage of territory, show a high consumption of Phosphorous and moderate consumption of Nitrogen (0.41 kton) and Potassium (1.32 kton). On the other hand, olive growing shows an almost similar consumption for all three fertilizers (7.88 kton for Nitrogen, 8.21 kton for Phosphorus and 6.01 kton Potassium).

**Figure 7:**
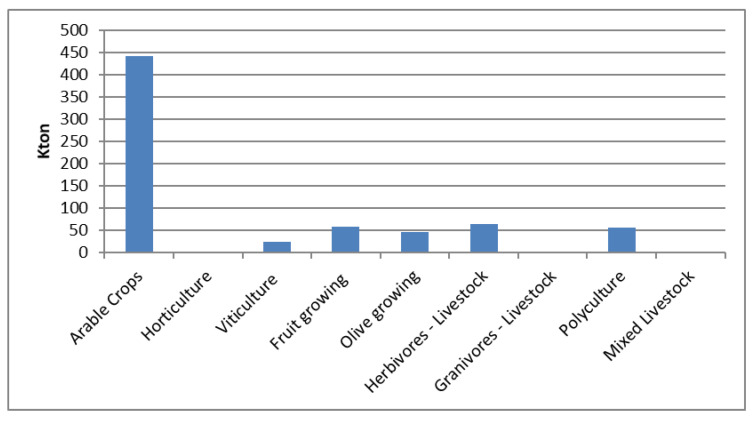
Waste production by farm category. Base year (2010).

As regards waste production (Figure 7) arable crops provide the highest values of dry matter (442.46 kton). Herbivorous livestock (64.66 kton of manure), fruit growing (59.27 kton of dry matter), polyculture (56.37 kton of dry matter) and olive growing (46.59 kton of dry matter) mixed livestock and granivorous produce a very low amount of waste (respectively 0.22 kton and 0.06 kton of manure) also produce small quantities while waste production from horticulture is negligible.

In the calibration runs, CO_2eq_ emissions from combustion processes are entirely determined by fossil fuel consumption for agricultural activities, with a total amount of CO_2eq_ of 131 ton for the base year, the highest contribution being provided by arable crops (61 ton of CO_2eq_) while the lowest by granivorous livestock (0.3 ton of CO_2eq_).

**Figure 8:**
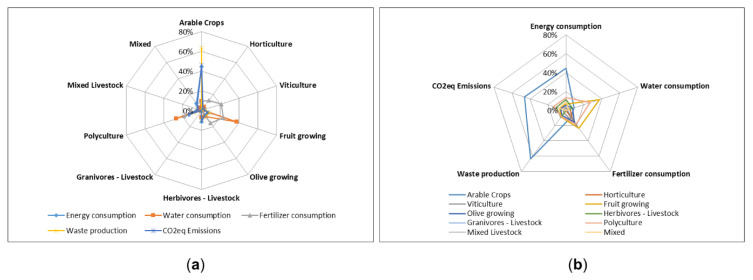
(**a**) Consumption and emissions distribution by type of agricultural activity (**b**) Weight of agricultural activities in resource consumption and emission production.

Figure 8 provides an overview of the distribution of the different commodities (energy consumption, water consumption, fertilizers consumption, waste production and CO_2eq_ emission production) by farming activity.

In the two polar maps of Figure 8, energy and fertilizer consumption were aggregated. Arable crops represent the category with the highest energy consumption (44%), associated CO_2eq_ emissions and waste production (64%). Fruit growing has the greatest weight in both water (37%) and fertilizer consumption (23%). Polyculture is the second category for water consumption (27%) and viticulture for fertilizer consumption (21%). Granivorous show the lowest energy consumption (0.3%), associated CO_2eq_ emissions (0.3%) and waste production (0.1%).

The calibration of the β version of the TIMES-WEF model highlights the differences between the various agricultural activities in terms of resources used and output, confirming the validity of the modeling approach and preparing a solid reference structure for the implementation of the scenarios.

## 4. Conclusions

This paper presents the fundamental steps for the implementation of a modeling platform that integrates the water-energy-food nexus approach into the energy system modeling framework, with the aim to investigate the impacts of climate change and EU policies on the agricultural system.

In fact, agriculture plays a crucial role in achieving sustainable development goals and climate targets. The rational use of soil, water and energy is essential to ensure the well-being of the population and adequate food production, while the adoption of sustainable agricultural practices can help mitigating the effects of climate crisis and supporting adaptation to extreme weather events.

The Nexus approach, increasingly used for an integrated vision of the main challenges of sustainability, allows highlighting the interdependencies between the three key variables Water-Energy-Food and to understand how they are influenced by climate change and the policies in place. The study goes beyond the state of the art [26] by designing, implementing, and validating a modeling approach based on energy system analysis, widely used in policy assessment.

A broad scientific debate is underway about the choice of modeling tools and indicators for defining policy strategies and measuring progress toward the achievement of sustainable goals. In fact, the selection of indicators can deeply influence the results of the monitoring [82] and the implementation of SDGs, requiring broader qualitative analyses [83].

In this context, the use of a modeling framework based on the IEA-ETSAP methodology, explicitly designed for long-term energy-environment analyses, to design the least-cost pathways for a sustainable development, allows ensuring transparency in the basic assumptions and a high detail in the identification of the possible strategies.

The integration of the nexus approach in a comprehensive partial equilibrium model based on the ETSAP-TIMES structure makes possible to set up a robust platform to identify the optimal allocation of energy and material resources in compliance with the EU strategic policy targets and to explore possible alternatives, measuring their effectiveness in terms of economic, energy and environmental indicators.

An innovative modeling approach based on land use as driving variable was adopted to develop the TIMES-WEF model whose database includes non-energy resources (water, fertilizers, pesticides) among the input commodities while food and biomass residuals represent the output commodities. The β version of the model was customized and calibrated on the agriculture system of the Basilicata Region, to enable its straightforward integration with the former TIMES Basilicata energy model [50] in a circular economy perspective.

Starting from the official European Union Classification [84], ten end-use categories representing the entire agri-food system were identified (arable crops, horticulture, viticulture, fruit growing, growing olive growing, polyculture, livestock herbivorous, granivorous livestock, mixed and mixed livestock) and the model database was set up accordingly.

The modeling approach was validated by calibrating the model β version and evaluating the congruence of the results to the statistics.

The next steps will concern, first, the optimization of the model under the Reference Scenario, representing a “status quo” development along the time horizon and, second, a scenario analysis to identify suited roadmaps for a sustainable development of the regional agricultural system.

The proposed innovative modeling framework applied to a regional energy and agriculture system can contribute to supporting decision makers in a complex governance of a system in which conflicting objectives must be faced. In particular, a thorough analysis of the water-energy-food system will allow enhancing the role of the agriculture and forestry sectors to achieve the national CO_2eq_ reduction and RES targets, evaluating the effectiveness of different mitigation strategies.

At the same time, the assessment of the CAP at the local scale will provide insights to coordinate the implementation of policies at local and national level with a long-term perspective.

## Figures and Tables

**Table 1:** Summary of main methods used in the literature to address the Nexus.

Type	Brief Description	Examples
Computable General Equilibrium (CGE)	Used for long-term simulations, CGE models analyze the economic implications of policies (e.g., CO_2_ tax), assuming that all markets are in equilibrium and not considering the technological details	GEM-E3 [31], GTAP [32] and IMPACT [33].
Econometrics models	Oriented to test economic theory through empirical evidence, they currently include open and growth-based macro econometric models, with trend/analysis of time series data on a higher level of aggregation. Their main limitation lies in the strong dependence on data	E3ME [34] and IREDSS [35].
Input-output models	Suitable for short-term assessment of policies, as they can only provide a static image of the economic structure based on historical data illustrating sectoral production techniques describing the total flow of goods and services of an economic system in terms of production, added value and specific technical input/output coefficients.	[36].
Partial Equilibrium/Optimization	Used to support the decision-making process by providing policy makers with detailed information on technologies and resources on both the demand and supply sides. Partial equilibrium models are characterized by a high technology detail both in the supply and demand side and define the optimal set of technological choices to achieve multiple objectives at the minimum feasible cost in relation to predefined exogenous constraints.	POLES [37]; MARKAL/TIMES [26,27,28,29,30,31,32,33,34,35,36,37,38].
Simulation	They provide a descriptive and quantitative image of energy conversion and demand based on drivers and technical data exogenously, in order to model the decision-making process.	LEAP [39] and BUENAS [40].
GIS-based tools	Mathematical models for the representation of georeferenceable variables. They are used to transfer on a larger scale the assessments of the consumption of energy flows or other resources referred to the local scale	[41]

**Table 2:** List of data source and type of information provided.

Data Source	Type of Information Provided
ISTAT Agricultural Census [63]	Hectares of used agricultural area by type of farming; hectares of total agricultural area; annual water consumptions.
Regional Environmental Energy Plan–PIEAR [64]	Energy demand of Agriculture (diesel oil, electricity, and natural gas).
Annual RICA Survey (National source of European Farm Accountancy Data Network (FADN)) [65]	Annual micro data on surveyed farm: energy consumption (diesel, natural gas, and electricity), use of fertilizers, production (crops and cattle), fixed and variable production costs.
Local Chambers of Commerce [66,67]	Prices applied on a local scale to fertilizers and agricultural diesel.
Local Reclamation Consortium [68]	Agricultural water prices.
Ministry of Economic Development [69]	Natural gas prices.
Energy Service System Operator (GSE) [70]	Electricity prices.

**Table 3:** Energy Consumption in Agriculture for the Basilicata Region (PIEAR data).

Energy Fuel	PJ
Electricity	0.227
Natural gas	0.037
Diesel	1.741

**Table 4:** Percentage breakdown of energy consumption.

Type of Farming	Diesel (PJ)	Natural Gas	Electricity
Arable Crops	46.0%	58.2%	30.5%
Horticulture	2.6%	0.3%	3.2%
Viticulture-PermCrops_1	2.9%	6.0%	1.9%
Fruit growing-PermCrops_2	6.9%	14.2%	4.6%
Olive growing-PermCrops_3	5.5%	11.4%	3.7%
Herbivores Livestock	10.7%	9.6%	18.6%
Granivorous Livestock	0.3%	0%	0.6%
Polyculture	14.1%	0.4%	9.5%
Mixed Livestock	3.5%	0%	8.0%
Mixed	7.5%	0%	19.3%

**Table 5:** Water consumption by type of farming.

Type of Farming	M^3^/ton	M^3^/LSU	M^3^/ha
Arable Crops	15		
Horticulture	40		
Viticulture-PermCrops_1	48		
Fruit growing-PermCrops_2	272		
Olive growing-PermCrops_3	172		
Herbivores Livestock		70	
Granivores-Livestock		0	
Polyculture	156		
Mixed Livestock		436	
Mixed			144

**Table 6:** Consumption of fertilizers per hectare by type of crop.

Type of Farming	N (ton/ha)	P (ton/ha)	K (ton/ha)
Arable Crops	0.00157	0.04424	0.00510
Horticulture	0.01340	0.04316	0.01210
Viticulture-PermCrops_1	0.03110	0.05621	0.02763
Fruit growing-PermCrops_2	0.03351	0.06969	0.02525
Olive growing-PermCrops_3	0.03047	0.03175	0.02323
Polyculture	0.01900	0.05988	0.02074

**Table 7:** Consumption of fertilizers per ton of product and by type of crop.

Type of Farming	N (ton of N/ton of crop)	P (ton of P/ton of crop)	K (ton of K/ton of crop)
Arable Crops	0.0005	0.0153	0.0018
Horticulture	0.0236	0.0760	0.0213
Viticulture-PermCrops_1	0.2219	0.4011	0.1971
Fruit growing-PermCrops_2	0.0506	0.1052	0.0381
Olive growing-PermCrops_3	0.1471	0.1533	0.1121
Polyculture	0.0227	0.0714	0.0247

**Table 8:** Production and operating costs of agricultural activities.

Type of Farming	Unit of Measure	Production	Fixed Costs (Euro/ton or Euro/LSU)	Variable Costs (Euro/ton or Euro/LSU)
Arable Crops	ton	749,387	34	89
Horticulture	ton	146,729	55	930
Viticulture-PermCrops_1	ton	36,229	92	206
Fruit growing-PermCrops_2	ton	171,283	88	155
Olive growing-PermCrops_3	ton	53,555	250	545
Herbivores Livestock	LSU	127,693	323	589
Granivorous Livestock	LSU	20,096	255	699
Polyculture	ton	21,6746	61	168
Mixed Livestock	LSU	7691	277	1267

**Table 9:** Waste production by crops and livestock type.

Type of Farming	Straw Production		Manure Production
	Ton/ha	Ton/ton of Production	Ton/LSU
Arable Crops	1.99	0.6	
Horticulture	0	0	
Viticulture-PermCrops_1	2.15	0.65	
Fruit growing-PermCrops_2	2.20	0.35	
Olive growing-PermCrops_3	2.16	0.87	
Herbivores Livestock			0.53
Granivores-Livestock			0.03
Polyculture	2.13	0.26	
Mixed Livestock			0.1
